# Severe Acute Respiratory Syndrome: Relapse? Hospital Infection?

**DOI:** 10.3201/eid0909.030395

**Published:** 2003-09

**Authors:** Owen Tak-Yin Tsang, Tai-Nin Chau, Kin-Wing Choi, Eugene Yuk-Keung Tso, Wilina Lim, Ming-Chi Chiu, Wing-Lok Tong, Po-Oi Lee, Bosco Hoi Shiu Lam, Tak-Keung Ng, Jak-Yiu Lai, Wai-Cho Yu, Sik-To Lai

**Affiliations:** * Princess Margaret Hospital, Hong Kong; † Public Health Laboratory Centre, Hong Kong

**Keywords:** Keywords: Severe acute respiratory syndrome, SARS, coronavirus

**To the** **Editor**: Severe acute respiratory syndrome (SARS) is an emerging infectious disease worldwide, and relapsing SARS is a major concern. We encountered a 60-year-old woman who was admitted to the Princess Margaret Hospital in Hong Kong on March 29, 2003, with a fever of 39°C, chills, cough, malaise, and sore throat for 2 days before admission. She had no history of travel within 2 weeks of admission. She also had no close contact with patients who had a diagnosis of suspected or confirmed SARS. Chest radiograph on admission indicated consolidation over the right middle zone. In accordance with the diagnostic criteria proposed by the World Health Organization (WHO), this patient’s condition was diagnosed as SARS in view of her symptoms, temperature, and chest radiograph findings (*1*).

Standard microbiologic investigations to exclude common respiratory virus and bacterium for community-acquired pneumonia, including *Mycobacterium tuberculosis*, were negative in our patient. Reverse transcriptase–polymerase chain reaction (RT-PCR) of nasopharyngeal aspirate samples was negative for coronavirus twice. The coronavirus antibody titer was less than 1/25. The patient was initially treated with oral clarithromycin (500 mg twice a day) and intravenous amoxycillin-clavulanate combination (1.2 g three times a day). Despite the negative evidence for coronavirus infection, she was treated with intravenous ribavirin (24 mg/kg once a day) and hydrocortisone (10 mg/kg once a day) after 48 hours of antibiotics therapy (*2*). The patient’s symptoms were relieved, and she remained afebrile 3 days after admission. Tolerance for medication was good except for a moderate degree of hemolytic anemia (her hemoglobin level dropped to 9.1 g/dL) and hypokalemia that developed during treatment. On day 15, the chest radiography was clear. The patient was discharged after 3 weeks of hospital stay.

The patient attended outpatient clinic on day 35, complaining of exertional dyspnea, low-grade fever, and malaise since her discharge. Her chest radiography showed extensive shadowing. Computer tomographic scan of the thorax indicated widespread ground-glass shadowing in both lung fields, which was especially prominent at left lower and lingular lobes. Her hemoglobin level had dropped further to 8.4 g/dL. Sputum culture yielded substantial growth of methicillin-sensitive *Staphylococcus aureus* and *Pseudomonas aeruginosa*. RT-PCR results of throat and nasal swabs were positive twice for coronavirus, but coronavirus cultures from these areas were negative. One month after onset, her coronavirus antibody titer was 200. In view of possible relapse of SARS, she was treated with oral ribavirin (1,200 mg a day) and lopinavir (133.3 mg a day)/ritonavir (33.3 mg) combination (3 capsules twice a day) in addition to intravenous piperacillin/tazobactam combination. The patient was afebrile, and symptoms improved 3 days after admission. Serial chest radiograph showed gradual resolution of shadowing. Subsequent RT-PCR and sputum culture were negative.

This case illustrates several important issues regarding problems of infection control, diagnosis, and management of SARS. As the definition of SARS is nonspecific, patients with upper respiratory infection or community-acquired pneumonia could be mislabeled as having SARS. Accommodating confirmed SARS patients and patients mislabeled as having SARS in the same facility may be disastrous. Unfortunately, isolating every single case is impossible, particularly when a large number of patients are admitted. Our patient may have acquired the disease after admission since she was placed in the same ward with other patients confirmed to have SARS. For this reason, special cohorting of SARS patients with closely related signs and symptoms should be strictly implemented at admission. Since fever is the most common feature of SARS, isolating febrile cases with respiratory or gastrointestinal symptoms may be appropriate. Even patients with fever alone should be quarantined since the other symptoms of SARS may not be clinically obvious. Secondly, the sensitivity of diagnosing a coronavirus infection on admission is only 32% to 50% by nasopharyngeal RT-PCR test (*3*,*4*). Many infected cases will be missed as a result. Our patient may have had a relapse of disease during her second admission, although she had positive RT-PCR and antibody surge only 1 month after onset. However, we could not conclude whether the first RT-PCR on admission was a false negative or whether the patient acquired coronavirus infection in the hospital. Our study showed that sensitivity for diagnosing coronavirus infection could be increased by performing RT-PCR on samples from different parts of the body (*4*). Unfortunately, these samples were not taken from our patient. Furthermore, the chest infection with organisms recovered from her sputum could be the sole reason for her second admission, especially when her immune system was weakened by the administration of a high-dose steroid. The presence of genetic material for coronavirus from her nasal cavity and throat might not suggest that the virus is active. The absence of coronavirus growth in this patient might indicate that the virus is no longer viable, although the culture technique itself might not be sensitive enough to justify this claim. Therefore, further refinement of the diagnostic techniques for SARS is essential, especially for diagnosis during early onset. Thirdly, giving treatment to a patient without a legitimate diagnosis may be inappropriate, especially when the treatment carries substantial adverse effects, as illustrated in our patient, and a universally accepted therapy has not been available. Whether lopinavir/ritonavir combination is the key to a cure remains to be clarified, despite the satisfactory response that we observed, since the clinical and radiologic improvement in our patient might be the natural course of the disease.
